# SPAE-YOLOv8 for Onboard Real-Time Perception: Lightweight Small UAV Detection from Air-to-Air Perspectives

**DOI:** 10.3390/s26113424

**Published:** 2026-05-28

**Authors:** Rushang Zhang, Xiaogang Fu

**Affiliations:** School of Electrical Engineering, Shanghai DianJi University, Shanghai 201306, China; 24600001010504@st.sdju.edu.cn

**Keywords:** air-to-air UAV recognition, small target, lightweight, YOLOv8

## Abstract

The increasing use of UAVs has raised concerns regarding public safety and airspace security. To address air-to-air micro-UAV detection with cluttered backgrounds, tiny targets, and diverse viewing angles, this paper develops SPAE-YOLOv8, a lightweight detector based on YOLOv8n. SPAE consists of four core designs: SIoU loss, P2 shallow feature layer, ADown adaptive downsampling, and Efficient_UAVDet lightweight detection head. These modules improve small-target representation and reduce model size. In this paper, lightweight refers to the combination of parameter count, storage volume and inference speed. On the Det-Fly dataset, the proposed method achieves an mAP@0.5 of 0.922, outperforming YOLOv8n by 7.2 percentage points while reducing total parameters by 30%. We conduct independent training and testing on the DUT Anti-UAV dataset and obtain an mAP@0.5 of 0.906. Cross-dataset testing is further carried out on the more challenging Anti-UAV300 dataset without additional fine-tuning to verify the generalization performance of the model. In real-world onboard deployment, the model is implemented on an Intel NUC11TNHi7 embedded UAV platform with OpenVINO acceleration and achieves 43.9 FPS at a resolution of 640×640, satisfying real-time inference requirements. The ablation results demonstrate the contribution of the proposed modules, providing an efficient lightweight solution for airborne monitoring and civil airspace security.

## 1. Introduction

With the continuous development of unmanned aerial vehicle (UAV) technologies, micro-UAVs have been increasingly applied in various fields, including military reconnaissance, civil surveying and mapping, and emergency rescue [1]. Concurrently, this proliferation has given rise to critical concerns such as unauthorized intrusions, airspace disruptions, and broader public safety risks [2]. Air-to-air micro-UAV detection, as a core technology for airspace situational awareness and threat prevention and control, can provide real-time target early warning for carrier aircraft platforms. Its detection accuracy and real-time performance are directly related to mission execution efficiency and platform security. Therefore, this task has attracted increasing attention from both academic researchers and industrial practitioners. Over the past few years, advancements in aviation technology have progressively extended UAV operational domains from mid-to-high altitudes into unstructured low-altitude airspace [3]. Such complex low-altitude environments have introduced novel challenges for UAV autonomous perception and collision avoidance systems. Currently, UAV obstacle sensing in low-altitude settings primarily employs two sensor categories: active sensors (e.g., millimeter-wave radar, LiDAR, and ultrasonic devices) and passive visual sensing modalities including photoelectric and infrared technologies. Owing to their inherent merits of light weight, compact size, and rich environmental information, visual sensors have garnered growing interest in UAV collision avoidance research.

Traditional UAV visual detection algorithms typically depend on handcrafted feature extractors (e.g., HOG [4], SIFT [5]) paired with classifiers to realize target identification, yet these approaches suffer from limitations including limited feature robustness, poor adaptability to intricate backgrounds, and excessive computational costs, making them ill-suited for the demands of air-to-air dynamic detection scenarios. Recent developments in deep learning have catalyzed substantial advancements in target detection methodologies, which are broadly classified into one-stage and two-stage architectures according to their operational pipelines; represented by Faster R-CNN, two-stage detectors first generate candidate object regions before conducting classification and bounding box regression, and while such models deliver high detection precision, their sluggish inference speed and excessive parameter count render them incompatible with real-time detection constraints [6,7]. In contrast, one-stage algorithms directly perform target localization and classification, yielding notable gains in detection efficiency, among which the YOLO family of models has emerged as the dominant selection owing to its favorable trade-off between accuracy and inference speed [6,8,9]. As a robust, well-established, and readily deployable member of the YOLO series, YOLOv8 employs an anchor-free detection paradigm alongside the C2f feature extraction module, delivering exceptional performance across diverse target detection tasks, and its compact model footprint, flexible deployment options, and rapid inference speed render it particularly well-adapted for target detection in UAV-captured aerial images [7]. Nevertheless, the model exhibits notable shortcomings when applied to air-to-air small target detection: the low pixel occupancy of small targets impedes the extraction of meaningful information by deep feature maps, inefficient cross-scale feature fusion hinders the effective propagation of multi-level feature information, fine-grained details of small targets are prone to erosion during successive downsampling operations, and additionally, the redundant structure of the detection head complicates its adaptation to the computational limitations of airborne embedded systems [10].

In light of the above considerations, this work adopts YOLOv8n as the foundational framework and implements tailored modifications to its network architecture from the UAV perspective. YOLOv8n was selected as the baseline architecture because it is a representative and widely adopted lightweight YOLO detector with a mature implementation, stable training pipeline, and clear modular structure. These characteristics provide a reliable and transparent foundation for task-oriented architectural optimization in air-to-air micro-UAV detection. Moreover, using YOLOv8n as the baseline allows the effectiveness of each proposed component to be analyzed more clearly through controlled ablation experiments. To ensure a fair and comprehensive evaluation against recent YOLO variants, YOLOv10n and YOLOv11n were also considered and evaluated under the same experimental settings. To tackle the above-mentioned challenges, this work proposes the SPAE-YOLOv8 enhanced algorithm through comprehensive multi-aspect optimization. The core contributions of this study are summarized below.

- We employ the SIoU loss function to replace the original regression loss of YOLOv8, which optimizes the prediction of bounding boxes and significantly improves the localization precision for small targets.
- We introduce the P2 shallow feature layer to supplement the missing fine-grained details in deep feature extraction, thereby enhancing the feature representation capability of the model for small objects.
- We embed the ADown adaptive downsampling module and design a novel Efficient_UAVDet detection head based on grouped convolution. This design streamlines the network structure, drastically reduces computational overhead, accelerates inference speed, lowers network parameters and computational complexity, and effectively reduces feature information loss for small targets. Collectively, these improvements achieve a favorable balance between detection accuracy and real-time inference efficiency.

The remainder of this paper is organized as follows. Section 2 reviews related studies on small-UAV detection, lightweight network design, and air-to-air perception datasets. Section 3 presents the proposed SPAE-YOLOv8 framework and its main architectural components. Section 4 provides the experimental evaluation, including comparison experiments, ablation studies, cross-dataset validation, and onboard deployment tests. Finally, Section 5 concludes the paper and discusses future research directions.

## 2. Related Works

### 2.1. Small Object Detection and Lightweight Networks

Airborne moving targets typically present substantial scale variations, and this characteristic is especially pronounced in air-to-air aerial images [11]. Traditional detection methods usually utilize image pyramids to cope with the challenges arising from scale instabilities. Regarding small target detection, two mainstream approaches are currently adopted: one entails constructing a multi-branch network architecture, while the other emphasizes extracting unique characteristics of tiny objects via multi-level feature fusion. Both strategies aim to minimize information degradation during feature extraction to achieve superior detection performance. The former approach leverages multi-scale features from different network depths to match targets of varying scales, while the latter enhances small-target detection by fusing high-resolution positional details from shallow layers with high-level semantic information from deep layers via cross-layer interactions in the fusion stage [12]. In recent years, to tackle the notable scale discrepancies of small targets in air-to-air and UAV contexts, researchers have conducted in-depth explorations into multi-branch network architectures and multi-level feature fusion strategies. Concerning multi-branch network design, relevant studies typically integrate multiple parallel prediction branches or scale-specific detection heads into the detection framework, allocating distinct branches to manage targets within defined scale intervals and consequently improving the recall rate for small objects. Ning et al. [11] refined the YOLOv8 model for UAV-oriented detection scenarios by embedding a self-attention module into a newly built shallow detection head, which boosted the model’s small target detection capability and delivered encouraging results on the UAV small target dataset. Xu et al. [13] addressed the problem of uneven target size distribution in UAV imagery by introducing a high-resolution detection branch, allowing shallow-layer features to directly participate in target prediction and markedly elevating the detection precision of small objects.

In the aspect of multi-level feature fusion, recent research has paid more attention to the sufficient interaction of cross-layer feature information. Focusing on receptive field expansion, Li et al. [14] integrated the RFCBAM attention-enhanced convolution block into the SOD-YOLO backbone, redesigning the downsampling strategy to improve small target feature extraction. By concentrating on the spatial characteristics within the receptive field, this approach breaks the constraints imposed by the parameter-sharing mechanism of convolutional kernels, thus enhancing the feature representation capability of the backbone network.

Qu et al. [15] mitigated information loss during downsampling by introducing SPD-Conv and devised a GCU module to reinforce cross-layer feature fusion, which notably elevated the small target detection precision and validated the efficacy of structural-level optimization in UAV small target detection tasks. Furthermore, Wang et al. [16] integrated WIoUv3 into the bounding box regression loss and proposed a more reasonable gradient gain assignment strategy via a dynamic non-monotonic mechanism. By effectively suppressing the gradient gain of both high- and low-quality samples, this method boosts the model’s localization accuracy and generalization capability [16]. Existing research indicates that multi-branch structures and multi-level feature fusion methods achieve exceptional performance in small target detection, and have thus become the prevailing technical frameworks for air-to-air small target detection. In the domain of mobile and edge computing, boosting inference efficiency and reducing latency has emerged as a core research direction, motivating scholars to investigate a diverse range of lightweight deep learning network architectures [11]. Studies on lightweight optimization have been conducted from the dimensions of backbone networks, convolution operators, detection heads, and the overall network structure. In terms of backbone networks, MobileNet [17], ShuffleNet [18] and GhostNet [19] are three representative lightweight neural network series in this field. At the convolution module level, researchers have introduced lightweight convolution modules to reduce redundant feature computation at the convolution operator layer. Cao et al. [20] adopted GhostConv to construct a lightweight feature extraction module in the YOLO framework. This design yields effective feature maps with minimal arithmetic resources, thereby substantially diminishing the model’s parameter footprint and operational overhead in UAV small target detection scenarios. For detection head optimization, Ren et al. [21] put forward a decoupled head with automatically adjustable channel counts customized to the model scale. By separating classification and regression tasks, this architecture enhances the network’s detection performance. Under strict computational constraints, a lightweight deformable convolution module is introduced to allow convolution to extract regions of interest across a wider range, thereby improving the precision of the target detection network [21]. Tan et al. [22] proposed BiFPN to support efficient multi-scale feature integration. They embedded classification and regression subnetworks that share parameters across different scales. This parameter sharing mechanism cuts down redundant parameters in the detection head, ensuring that its complexity grows non-linearly with the number of feature maps. This optimization further elevates the overall model’s computational efficiency.

In addition, Dai et al. [23] reduced branch redundancy by unifying the detection head structure, lowered computational complexity through decomposed attention, and improved feature utilization efficiency via sparse spatial modeling. This design provides a valuable reference for building high-efficiency target detection models in resource-limited scenarios, including mobile terminals, edge computing devices, and UAVs.

These studies demonstrate that collaborative optimization from both module and architecture perspectives can improve model efficiency and practicability under hardware-limited conditions, while retaining the fundamental detection principle. Representative approaches are summarized in Table 1.

### 2.2. UAV Detection Datasets

In the research of object detection and segmentation for UAV-borne sensing, dataset construction has gradually formed a multi-dimensional and scenario-oriented system. According to the observation perspective and target type, UAV-related datasets can be divided into two categories: “air-to-air UAV target detection datasets” (UAV as the detected target) and “ground-target detection datasets from UAV perspective” (UAV as the imaging platform). These two types of datasets have completely different characteristics and application scenarios.

For air-to-air UAV target detection datasets, the research goal is to detect flying UAVs, which is also the core scenario of this paper. The early Aircraft dataset [24] explored the detection of aerial flying targets, but it is not open-source and lacks specialized UAV targets. The Det-Fly dataset [25] is a representative public dataset for micro quadrotor UAV detection with extremely small targets. The Avian-Airborne dataset [26] covers aircraft, birds, and UAVs, supporting multi-category aerial target perception. The ARD100 dataset [27] provides dual-modal annotations of RGB and motion difference images for tiny UAVs. The MMFW fixed-wing dataset [28] is designed for fixed-wing UAVs and contains multi-sensor data. In addition, the DUT Anti-UAV dataset [29] and Anti-UAV300 dataset [30] are typical anti-UAV evaluation benchmarks, which contain challenging samples such as small scales, complex backgrounds, motion blur, and occlusion, and are highly consistent with the air-to-air UAV detection task in this paper.

For ground-target detection datasets from a UAV perspective, the UAV only serves as an aerial imaging platform, and the detection targets are ground objects such as vehicles, pedestrians, and buildings. The classic semantic segmentation benchmark dataset UAVid [31] contains 30 groups of 4K resolution oblique-view image sequences with 8 categories of urban street-scene annotations, laying an evaluation foundation for UAV-based urban semantic segmentation tasks. The AU-AIR dataset [32] fuses multi-modal data including RGB, GPS, and IMU, which can support multi-task research such as semantic segmentation and instance segmentation. The VisDrone dataset [33] constructed by the AISKYEYE team of Tianjin University has become a global benchmark. Collected from 14 cities, it includes more than 260,000 frames and 2.6 million annotated targets, covering urban and rural environments, sunny/rainy/foggy weather, and various target forms [34]. The dataset provides refined annotations for attributes such as occlusion and truncation, offering complete verification conditions for dense tiny-target detection and cross-scene generalization algorithms. In addition, datasets such as UAVDT and WTA/TLA focus on vehicle tracking and energy facility segmentation in vertical top-view scenarios, forming a complete UAV dataset ecosystem from general to dedicated and from single-modal to multi-modal.

However, existing resources still have obvious limitations. Most datasets are designed for ground-target detection from UAV perspective, not specifically for detecting flying UAVs. The number of real air-to-air UAV target datasets remains insufficient, and most suffer from single backgrounds or viewpoints, lacking multi-angle observations and complex scenes. A detailed comparison of representative datasets is presented in Table 2.

## 3. Materials and Methods

Ultralytics introduced YOLOv8 in 2023 as an advanced object detection framework, building on YOLOv5 with targeted architectural enhancements [35,36]. Key modifications include replacing the backbone’s C3 module with the more efficient C2f block, simplifying convolutional layers in the neck’s upsampling stage, and converting the original coupled detection head into a decoupled design [37,38]. The model retains the SPPF module and network-width scaling strategy from YOLOv5 and offers multiple variants optimized for different computational resources and detection requirements [39].

The network is structured into four key parts: input, backbone, neck, and detection head [40]. The input pipeline handles raw data preprocessing, including Mosaic augmentation, adaptive image resizing, and dynamic anchor optimization, which increase training diversity and accelerate convergence. The backbone, based on an evolved CSPDarknet, stacks convolutional and C2f modules to extract high-dimensional semantic features, while residual connections and bottleneck structures mitigate the problems of gradient disappearance and gradient explosion in deep network training. The neck integrates FPN and PAN structures to perform multi-scale feature fusion, combining multi-level features from the backbone to enhance the representational power of fused feature maps [41]. The detection head utilizes an anchor-free paradigm, separating the multi-scale neck outputs into classification and regression branches, which are trained using Varifocal Loss (VFL) together with Complete IoU (CIoU) and Distribution Focal Loss (DFL) [42,43,44].

**Figure 1:**
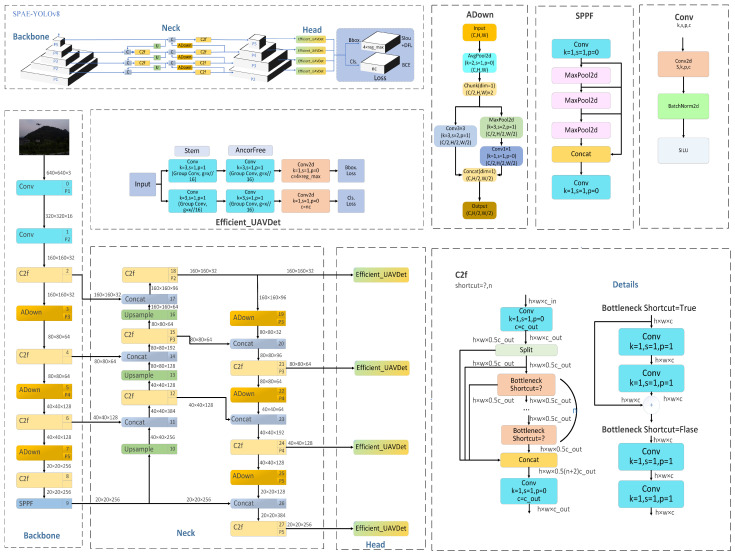
Architecture of the SPAE-YOLOv8 model with modular decomposition into backbone and detection head. Different colors indicate different functional modules, and arrows represent the feature-flow directions and connection relationships.

To address the limitations of YOLOv8n including insufficient detection accuracy, low feature fusion efficiency, and an inadequate trade-off between inference speed and precision in air-to-air micro-UAV small target detection, an improved lightweight YOLOv8n scheme optimized for small targets is proposed. The core lies in full-link optimization from four dimensions, feature level expansion, feature fusion mechanism, downsampling strategy, and detection head structure, so as to strengthen the applicability and performance of small target detection while maintaining lightweight characteristics. The proposed algorithm’s overall structure is depicted in Figure 1.

### 3.1. SIoU Loss Function

**Figure 2:**
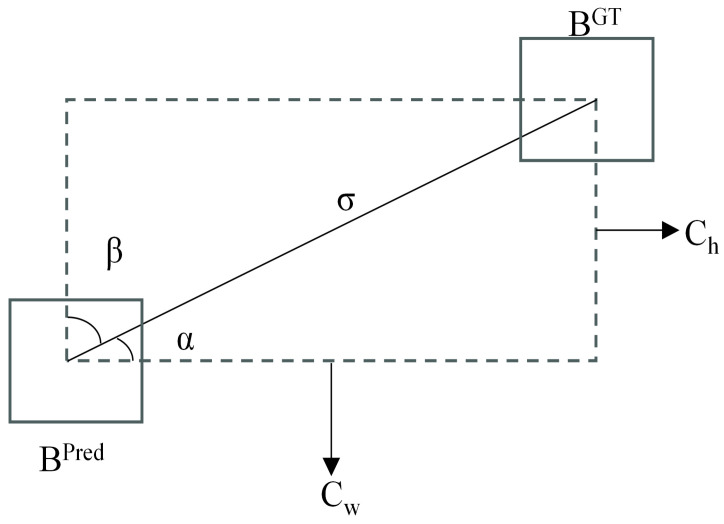
Center-point connections between model-predicted and annotated boxes.

The native CIoU loss employed by YOLOv8 merely takes intersection area, center offset, and aspect ratio into account during bounding box regression. To achieve more precise localization, the SIoU loss function is adopted instead, as it accounts for additional factors including angle, distance, and shape, allowing the network to better adjust predicted boxes. In this study, the SIoU loss replaces the default CIoU loss to improve the model’s ability to distinguish small UAV targets from complex backgrounds. Figure 2 illustrates the spatial relationship between the centers of predicted boxes and ground truth boxes. The SIoU loss is composed of three main components, which collectively guide the optimization of bounding box predictions.

(1) The angular loss, denoted as Λ, is determined by the minimum angle between the center-connecting line of the predicted box and the reference box, and the *x*-axis and *y*-axis. Its mathematical formulation is presented below.

(1) $$\Lambda =1-2\sin^{2}\left(\sin^{-1}\left(\frac{c_{h}}{\sigma }\right)-\frac{\pi }{4}\right)$$

(2) Denoted as Δ, it characterizes the separation between the central points, and its penalty exhibits a positive correlation with the angular cost. The contribution becomes more significant as α approaches π/4. Its mathematical formulation is provided below.

(2) $$\Delta =\sum_{t=x,y}1-e^{-\left(2-\Lambda \right)P_{t}}$$

(3) $$P_{x}={\left(\frac{B_{cx}^{gt}-B_{cx}}{C_{w}}\right)}^{2}$$

(4) $$P_{y}={\left(\frac{B_{cy}^{gt}-B_{cy}}{C_{h}}\right)}^{2}$$

In the formulas, $B_{cx}^{gt}$ and $B_{cy}^{gt}$ stand for the central position of the real target box, while $B_{cx}$ and $B_{cy}$ correspond to the predicted box. $C_{w}$ and $C_{h}$ denote the width and height of the smallest rectangle enclosing both boxes.

(3) The shape loss, denoted as Ω, quantifies the ratio difference of width and height between model outputs and real object boxes, helping predictions better match the actual object shapes and enhancing detection stability. Its mathematical expression is given below.

(5) $$\Omega =\sum_{t=w,h}{\left(1-e^{w_{t}}\right)}^{\theta }$$

(6) $$w_{w}=\frac{\left|w-w^{gt}\right|}{Max\left(w,w^{gt}\right)}$$

(7) $$w_{h}=\frac{\left|h-h^{gt}\right|}{Max\left(h,h^{gt}\right)}$$

In this case, *w* and *h* indicate the size of the regression box, $w^{gt}$ and $h^{gt}$ stand for the dimensions of the real target, and θ refers to the attention weight assigned to the shape loss. Relevant equations are displayed in (5) to (7).

### 3.2. P2 Small Target Detection Layer Expansion

For an input resolution of 640 × 640 pixels, the smallest detection head in the YOLOv8n model corresponds to the P3 feature map, which covers a receptive field of 80 × 80 pixels. For micro-UAV targets smaller than 80 pixels, it is particularly challenging to extract effective feature information due to the limited area of their two-dimensional information. To address this issue, an additional P2 feature layer is integrated into the backbone, as illustrated in Figure 1, aiming to enhance the network’s ability to capture tiny targets and improve detection accuracy in complex environments.

In the output of the C2f module, the new P2 layer has a scale of 160 × 160 pixels. This layer is generated by using a 1 × 1 convolution to reduce dimensionality and conducting feature calibration on the features from the third backbone layer, preserving richer shallow details and positional information. The P2 layer is then fused with the upsampled features from the neck to build a multi-scale feature pyramid (“P2-P3-P4-P5”), enabling the network to more effectively capture fine-grained details of small UAV targets while boosting both recall and localization performance.

### 3.3. ADown Refined Downsampling Module

For air-to-air micro-UAV detection, repeated downsampling may weaken the boundary and texture cues of tiny targets, especially when the UAV occupies only a small number of pixels in the image. To reduce feature degradation during spatial resolution reduction, the ADown module is introduced as a lightweight refined downsampling block. Instead of relying on a single stride-2 convolution, ADown adopts a dual-branch structure to preserve complementary feature responses while reducing computational cost.

In the practical implementation of ADown, the input feature tensor is first processed by an average pooling operation, which helps retain local contextual information before subsequent downsampling. The resulting feature tensor is then split into two equal parts along the channel dimension, denoted as $x_{1}$ and $x_{2}$. For the $x_{1}$ branch, a 3×3 convolution with stride 2 is used to perform spatial downsampling and feature extraction. For the $x_{2}$ branch, a 3×3 max-pooling operation is first applied to emphasize salient local responses, followed by a 1×1 convolution for channel refinement.

The outputs of the two branches have the same spatial resolution and are concatenated along the channel dimension. As a result, the ADown module combines the smoothing effect of average pooling, the salient-response enhancement of max pooling, and the representation ability of convolutional operations. This design enables the network to obtain richer downsampled features while maintaining a compact structure. The structural schematic is illustrated in Figure 1.

The ADown module employs 3×3 and 1×1 convolutional kernels to control the parameter count and computational complexity. By splitting the feature channels before downsampling, the module reduces redundant convolutional computation compared with conventional stride-2 convolution. Given an input feature map with the dimension of h×w×c, the downsampled output has the size of h/2×w/2×c, where *h* and *w* denote the height and width, respectively, and *c* represents the channel number. The parameter counts $P_{a}$ and $P_{c}$, as well as the computational costs $F_{a}$ and $F_{c}$ associated with the ADown module and the 3×3 convolution with stride 2, are presented in Equations (8) and (9).

(8) $$\left\{\begin{array}{c} P_{a}=\frac{5}{2}c^{2}\\ F_{a}=\frac{5}{8}c^{2}\times h\times w \end{array}\right.$$

(9) $$\left\{\begin{array}{c} P_{c}=9c^{2}\\ F_{c}=\frac{9}{4}c^{2}\times h\times w \end{array}\right.$$

As reflected in Equations (8) and (9), the conventional 3×3 convolution with stride 2 for downsampling introduces 3.6 times more parameters and computational overhead than the presented ADown module. By significantly decreasing the parameter quantity and computational complexity compared with traditional downsampling convolution, the ADown module enables the network to maintain detection accuracy while effectively reducing computational consumption.

Although SPD-Conv has been reported as an effective downsampling strategy for micro-scale object detection by reducing spatial information loss, it rearranges spatial resolution into channel dimensions, which may increase channel expansion and memory access cost when embedded into a lightweight multi-scale detector. In contrast, ADown combines average pooling, max pooling, and convolutional branches to retain complementary downsampled features while reducing parameters and computational complexity. In the proposed framework, the P2 shallow feature layer is mainly responsible for enhancing fine-grained small-target representation, whereas ADown mainly serves as an efficiency-oriented downsampling module for model compression and onboard real-time inference. Therefore, ADown was selected to balance small-target feature preservation, model compactness, and real-time deployment requirements.

### 3.4. Efficient_UAVDet Head

Aiming at the problem that the original detection head of YOLOv8n uses ordinary 3×3 convolution, which results in high computational cost for airborne embedded systems, a stem feature enhancement module based on adaptive grouped convolution is designed.

The standard convolutions located before the regression and classification branches of the original detection head are substituted with channel-adaptive grouped convolution, where the group number *g* is dynamically adjusted according to the input channel count *x* of each individual branch. Here, *x* represents the total channel dimension of the input feature map corresponding to the respective detection head branch. The group number is calculated as follows:

(10) $$g=\left\lfloor \frac{x}{16}\right\rfloor$$

Based on this grouping, the core operation of the channel-adaptive grouped convolution can be formulated as:

(11) $$Y=\mathrm{GroupConv}\left(X,k=3,g\right)=\mathrm{Concat}{\left[\mathrm{Conv}(X_{i},k=3)\right]}_{i=1}^{g}$$

The input feature corresponding to the *i*-th group is denoted by $X_{i}$, with each group containing x/g channels. When the input and output channel numbers are equal, the parameter count of a single 3×3 grouped convolution is reduced from $9x^{2}$ to $9x^{2}/g$. This achieves a *g*-fold reduction in parameters for each convolutional layer, while two consecutive grouped convolution layers are employed to preserve feature extraction capability and maintain the lightweight property of the detection head.

**Figure 3:**
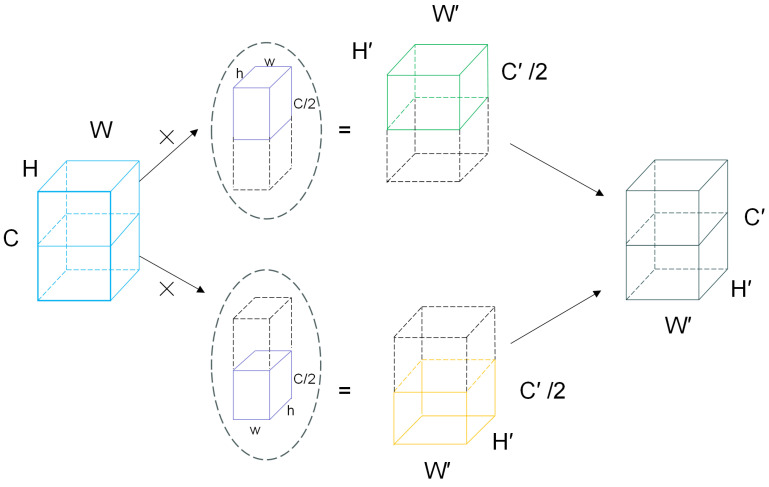
Schematic Diagram of Group Convolution’s Basic Operational Logic.

Figure 3 illustrates the principle of grouped convolution. The input feature map is divided into two groups along the channel axis, with each group containing half of the total channels. Each group is independently processed using a convolution kernel of the appropriate size, and the resulting outputs are then concatenated to form the final feature. This strategy lowers the parameter count to 1/g of a standard convolution, where *g* denotes the number of groups. In the detection head of this paper, a channel-adaptive version of grouped convolution is employed. Unlike the schematic example with a fixed group setting of g=2 shown in Figure 3, the number of groups here is dynamically computed according to the input channel count *x* in the detection head branch, using the formula $g=\left\lfloor \frac{x}{16}\right\rfloor$ (for example, if x=32, then g=2). The input features are split into *g* groups, convolved independently, and then concatenated, which not only preserves the lightweight advantage of grouped convolution but also adapts to varying channel scales across the multi-branch structure of the YOLOv8n detection head.

**Figure 4:**
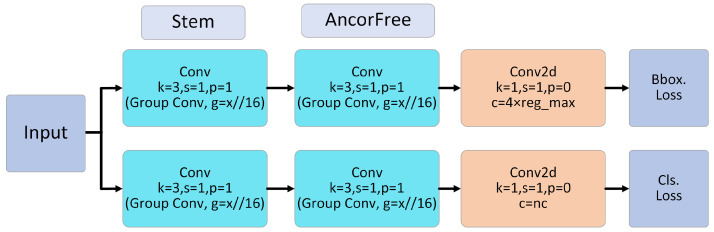
Structure of lightweight detection head based on adaptive group convolution.

Figure 4 illustrates the overall design of the stem feature enhancement module, which is implemented with two consecutive layers of 3×3 convolution using identical specifications. To maintain consistent feature dimensions, the input and output channel counts are aligned. By substituting standard convolution with the proposed channel-adaptive grouped convolution, the parameter count of each 3×3 convolutional layer is significantly reduced from $9x^{2}$ to $9x^{2}/g$. For instance, considering the P2 branch with an input of x=32 channels, setting g=2 results in the grouped convolution having only one-half of the parameters compared with conventional convolution. Additionally, stacking two layers of grouped convolution compensates for the limited feature extraction capability of a single layer, while multiple nonlinear transformations enhance the representation of target features and preserve the lightweight nature of the detection head.

Table 3 lists the input channel count *x*, group number *g*, and channels per group x/g for each branch of the YOLOv8n detection head. Here, *x* denotes the input channel number of each detection head branch, *g* denotes the number of groups used in grouped convolution, and x/g denotes the number of channels assigned to each group. Since the input channels of all detection heads are integer multiples of 16, the group number is calculated as $g=\left\lfloor \frac{x}{16}\right\rfloor$, thereby maintaining 16 channels in each group. The selection of 16 channels per group is further validated through a sensitivity analysis in Section 4.5.

## 4. Experimental Results and Analysis

### 4.1. Det-Fly Dataset

**Figure 5:**
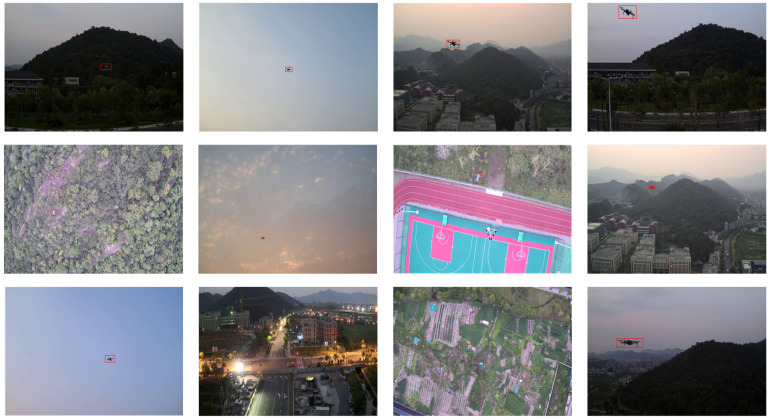
Sample images of the Det-Fly dataset (with author-annotated ground-truth for intuitive display). The red boxes indicate the ground-truth bounding boxes of UAV targets.

In this experiment, the public Det-Fly UAV detection dataset was adopted, which consists of 13,271 images of flying target UAVs (DJI Mavic series) captured by a DJI M210 UAV (SZ DJI Technology Co., Ltd., Shenzhen, China), with a resolution of 3840 × 2160 and an average target size accounting for 0.12% of the image. Its diverse scenarios make Det-Fly an ideal benchmark dataset for evaluating UAV detection models in various real-world conditions. This dataset includes UAV images under different weather conditions, viewing angles, and backgrounds in air-to-air scenarios, with complex backgrounds such as urban building clusters, cloud layers, and the sky. The dataset was split into training, validation, and test sets comprising 70%, 20%, and 10% of the total images, respectively, with several representative images shown in Figure 5.

### 4.2. Environment and Experimental Setup

All experiments were conducted on a high-performance workstation running Windows 10, equipped with an Intel i9-13900K CPU (Intel Corporation, Santa Clara, CA, USA), and an NVIDIA GeForce RTX 4090 GPU (NVIDIA Corporation, Santa Clara, CA, USA) with 24 GB of memory, and 32 GB of RAM. The software environment consisted of Python 3.8, PyTorch 1.13.0, cuDNN 8.5, and CUDA 11.7. Based on the original YOLOv8 backbone network, the architecture was further improved and optimized specifically for air-to-air UAV detection tasks. Ablation studies were performed to evaluate different design configurations and determine the optimal setup. The training parameters and other relevant settings are summarized in Table 4 to ensure both detection accuracy and computational efficiency.

The training parameters in Table 4 were selected to ensure stable convergence and fair comparison among different detection models. The input image size, imgsz, was set to 640 to preserve sufficient spatial details for small UAV targets while maintaining computational efficiency. The batch size was set to 32 according to the available GPU memory, providing stable gradient updates during training. The number of epochs was set to 200 to allow the model to reach convergence on the Det-Fly dataset. The number of workers was set to 4 to balance data loading efficiency and system stability.

SGD was adopted as the optimizer. The initial learning rate lr0 was set to 0.01, and the final learning rate factor lrf was set to 0.01 to gradually reduce the learning rate during training. The momentum was set to 0.937 to accelerate convergence and stabilize parameter updates, while weight_decay was set to 0.0005 to reduce overfitting and improve generalization ability. In addition, a warm-up strategy was applied during the first three epochs, with warmup_epochs set to 3 and warmup_momentum set to 0.8, to reduce training instability at the early stage. The close_mosaic parameter was set to 10, meaning that Mosaic augmentation was disabled during the last 10 epochs. This allows the model to fine-tune on images closer to the original data distribution, which is beneficial for improving the localization accuracy of small UAV targets. For YOLO-based models, the same training parameter settings were adopted to ensure fair comparison. For non-YOLO detectors such as RT-DETR and Faster R-CNN, model-specific recommended training configurations were used, while the dataset split, input resolution, hardware environment, and evaluation metrics were kept consistent.

### 4.3. Evaluation Metrics

In this paper, we adopt standard evaluation metrics for object detection, including Precision, Recall, mAP@0.5, mAP@0.5:0.95, FPS, and model size. Precision reflects the accuracy of model predictions, and Recall represents the ability to detect all real targets. mAP@0.5 and mAP@0.5:0.95 evaluate detection precision under different IoU thresholds. FPS characterizes real-time inference performance, and model size indicates the lightweight level for embedded deployment. In addition to FPS, detection latency is also reported in the onboard deployment experiment as the average processing time per image, which provides a more direct indication of the real-time response capability of the proposed model in micro-UAV applications.

To intuitively compare the overall efficiency of different models, we propose an Efficiency Score, defined as:

(12) $$\mathrm{Efficiency}\;\mathrm{Score}=\frac{\mathrm{mAP}\;@0.5\times \mathrm{FPS}}{\mathrm{Parameters}\;\left(M\right)}$$

This metric comprehensively balances detection accuracy, inference speed, and model complexity.

### 4.4. Comparison Experiments

To evaluate the performance of SPAE-YOLOv8, lightweight one-stage detectors, representative two-stage detectors, and end-to-end detectors were selected as comparison models. All models were evaluated on the same Det-Fly dataset split, with the same input resolution, hardware environment, and evaluation metrics. For YOLO-based models, the training configuration in Table 4 was adopted, while non-YOLO detectors such as RT-DETR and Faster R-CNN were trained using their model-specific recommended settings.

Although YOLOv8n was used as the architectural baseline for module-level improvement, recent lightweight YOLO variants such as YOLOv11n were also included to ensure that the proposed method is evaluated not only against the original baseline, but also against newer YOLO detectors. In addition, Faster R-CNN was included as a representative two-stage detector, and RT-DETR-R18 was included as a representative end-to-end detector, thereby broadening the comparative baseline beyond the one-stage detection paradigm. The corresponding performance results are presented in Table 5.

**Figure 6:**
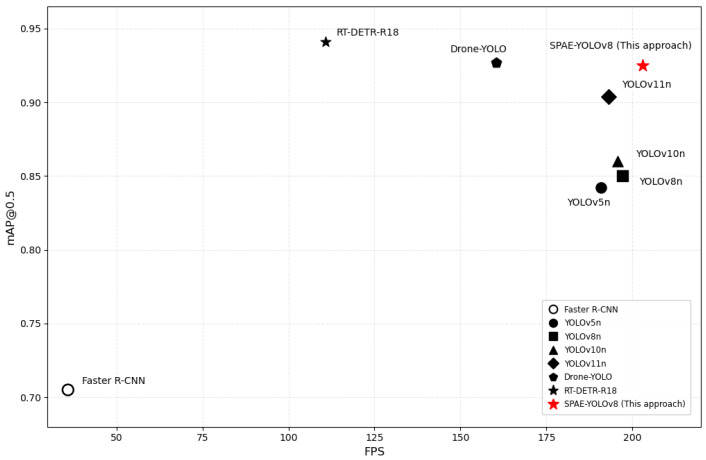
FPS versus mAP@0.5 scatter plot of all compared models.

Figure 6 intuitively illustrates the speed–accuracy trade-off of all compared models, where the proposed SPAE-YOLOv8 achieves the optimal balance with high mAP@0.5 and the highest FPS.

**Figure 7:**
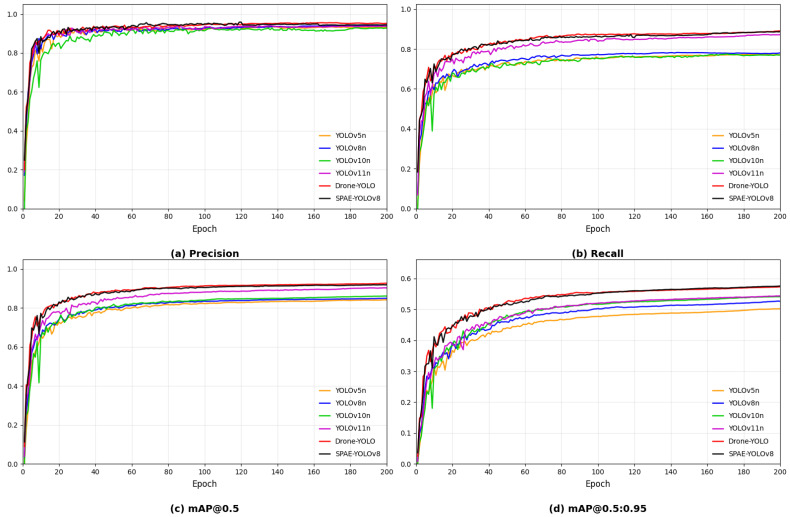
Training results within 200 epochs on the Det-Fly dataset validation set: (**a**) precision curve, (**b**) recall curve, (**c**) mAP@0.5 curve, and (**d**) mAP@0.5:0.95 curve.

As shown in Table 5, the SPAE-YOLOv8 method introduced in this study achieves an mAP@0.5:0.95 of 0.582, outperforming the lightweight YOLO-based models and Drone-YOLO, demonstrating its strong detection robustness for air-to-air small targets under various IoU thresholds. The training curves in Figure 7 further show that the proposed model maintains stable convergence and achieves consistently competitive validation performance during the 200-epoch training process.

For the newly added non-YOLO baselines, Faster R-CNN, as a representative two-stage detector, shows limited suitability for onboard small-UAV detection. Although it follows a region-proposal-based detection paradigm, it achieves only 0.705 mAP@0.5 and 0.326 mAP@0.5:0.95, while requiring 28.3 M parameters and a model size of 108.2 MB. Its inference speed is only 35.9 FPS, indicating that the two-stage framework has relatively high computational and storage costs for resource-constrained airborne deployment. RT-DETR-R18, as a representative end-to-end detector, achieves the highest mAP@0.5 of 0.941 and mAP@0.5:0.95 of 0.620, but it requires 19.9 M parameters and a model size of 38.6 MB, and its inference speed is reduced to 110.8 FPS. Therefore, although RT-DETR-R18 provides strong detection accuracy, its heavier model scale and lower inference speed make it less suitable for lightweight onboard real-time deployment.

In contrast, SPAE-YOLOv8 achieves a precision of 0.954 and a recall of 0.881, striking an effective balance between detection accuracy and recall for small UAV targets. In terms of lightweight performance, SPAE-YOLOv8 uses only 2.1 M parameters, and its model weight size is merely 4.3 MB, both of which are optimal among the compared models. Its inference speed reaches 203.0 FPS, far exceeding the 35.9 FPS of Faster R-CNN, the 110.8 FPS of RT-DETR-R18, and the 160.5 FPS of Drone-YOLO, meeting the real-time detection requirements of airborne equipment. Compared with the baseline YOLOv8n model, SPAE-YOLOv8 increases the mAP@0.5 by 7.2 percentage points and reduces the parameter count by 30%, fully verifying the effectiveness of multi-module integration and complementarity.

### 4.5. Modular Ablation Studies

To visually illustrate the performance differences of each proposed module, we conducted ablation studies based on YOLOv8n to verify the multi-module collaborative effects. The corresponding results are summarized in Table 6.

Based on the experimental results, the functional analysis of each improved module is presented as follows.

It should be noted that the proposed modules contribute to the final model from different aspects. The P2 small-target detection layer mainly improves detection accuracy and recall, while ADown and Efficient_UAVDet mainly contribute to parameter reduction, model compression, and inference acceleration. Therefore, not every module independently brings a significant accuracy gain; rather, the final model benefits from the complementary trade-off among accuracy, model complexity, and real-time performance.

After introducing the SIoU loss alone, the accuracy metrics of the model fluctuate slightly, while the recall is marginally improved. Specifically, the recall increases from 0.780 to 0.792, and mAP@0.5 slightly increases from 0.850 to 0.851, whereas mAP@0.5:0.95 decreases from 0.529 to 0.517. This indicates that SIoU can improve the localization tendency of some predicted boxes, but its independent contribution to the overall accuracy is limited. For air-to-air small UAV targets with weak texture and small pixel occupancy, improving the regression loss alone is insufficient to fully compensate for the lack of fine-grained feature representation.

Incorporating the P2 feature layer alone increases the recall from 0.780 to 0.886 and improves the mAP@0.5 by 7.5 percentage points. Meanwhile, mAP@0.5:0.95 increases from 0.529 to 0.576. These results suggest that shallow-level, high-resolution features enhance the model’s ability to capture fine-grained details of small objects, thereby improving detection performance. Furthermore, when the P2 layer is combined with the SIoU loss, the mAP@0.5 further reaches 0.929, and mAP@0.5:0.95 reaches 0.589, indicating that the two components can work collaboratively to achieve better optimization. This also shows that the P2 layer is the major source of accuracy improvement in the proposed framework.

After introducing the ADown downsampling module, the number of parameters decreases from 3.0 M to 2.6 M. However, the accuracy gain brought by ADown alone is marginal. The mAP@0.5 only increases from 0.850 to 0.854, with a gain of 0.004, while mAP@0.5:0.95 decreases from 0.529 to 0.524. This indicates that ADown mainly contributes to reducing model complexity and storage cost rather than significantly improving detection accuracy when used alone. The slight decrease in mAP@0.5:0.95 may be caused by information compression during lightweight downsampling, which can slightly affect precise localization under stricter IoU thresholds.

Replacing the original detection head with Efficient_UAVDet increases the inference speed from 197.2 FPS to 252.9 FPS and reduces the model size to 4.8 MB. The parameter count is also reduced from 3.0 M to 2.4 M. However, mAP@0.5 decreases from 0.850 to 0.846, and mAP@0.5:0.95 decreases from 0.529 to 0.521. Therefore, Efficient_UAVDet is mainly designed for model compression and inference acceleration rather than direct accuracy improvement. The slight accuracy degradation may be attributed to grouped convolutions, which reduce computational cost but also limit information interaction among different channel groups. For small UAV targets with weak visual features and strong background interference, insufficient cross-channel communication may slightly weaken the feature representation capability of the detection head.

To compensate for the minor accuracy loss caused by grouped convolutions, channel shuffle could be considered as a potential improvement strategy. It can enhance information exchange among different channel groups without significantly increasing convolutional parameters. However, channel shuffle also introduces additional channel reordering operations, which may increase memory access cost and inference latency on embedded platforms. Therefore, future work will further investigate whether channel shuffle can recover accuracy while preserving real-time inference efficiency.

When all proposed improvement modules are integrated into YOLOv8n, the model achieves an effective balance between accuracy and speed, with the mAP@0.5 reaching 0.922, mAP@0.5:0.95 reaching 0.582, the number of parameters being only 2.1 M, the model weight being merely 4.3 MB, and the inference speed hitting 203.0 FPS. Compared with the baseline YOLOv8n, the final model improves mAP@0.5 by 7.2 percentage points and mAP@0.5:0.95 by 5.3 percentage points, while reducing the parameter count by approximately 30%. These results demonstrate that the final performance gain is mainly produced by the complementary effects of different modules. The P2 layer enhances small-target feature representation, while ADown and Efficient_UAVDet improve lightweight deployment and inference efficiency. Therefore, the proposed model achieves a favorable trade-off among detection accuracy, model size, parameter count, and inference speed, making it suitable for real-time onboard deployment.

To further justify the setting of 16 channels per group in the proposed Efficient_UAVDet head, three different channel-per-group configurations were evaluated, as shown in Table 7.

A sensitivity analysis was conducted by comparing three channel-per-group settings, namely x/g=8, x/g=16, and x/g=32. Under the same training and validation settings, the corresponding group numbers for the P2, P3, P4, and P5 detection heads are 4/8/16/32, 2/4/8/16, and 1/2/4/8, respectively.

As shown in Table 7, setting x/g=8 yields the smallest model with 1.99 M parameters, 6.0 GFLOPs, and a model size of 4.1 MB. However, excessive grouping restricts inter-channel information exchange, resulting in slightly lower recall and mAP@0.5:0.95 than the x/g=16 setting, although it achieves the same mAP@0.5 of 0.922. When x/g=32, the grouped convolution becomes closer to standard convolution, which increases the parameter count to 2.19 M and the computational cost to 7.3 GFLOPs. However, this setting does not improve detection accuracy, with the mAP@0.5 decreasing to 0.918 and the FPS dropping to 196.4. In contrast, x/g=16 achieves the highest recall of 0.881, the highest mAP@0.5:0.95 of 0.582, and the best FPS of 203.0, while maintaining a compact model size of 4.3 MB. Therefore, 16 channels per group is selected as the final configuration because it provides the best trade-off among detection accuracy, model complexity, computational cost, and inference speed.

### 4.6. Algorithm Validation

**Figure 8:**
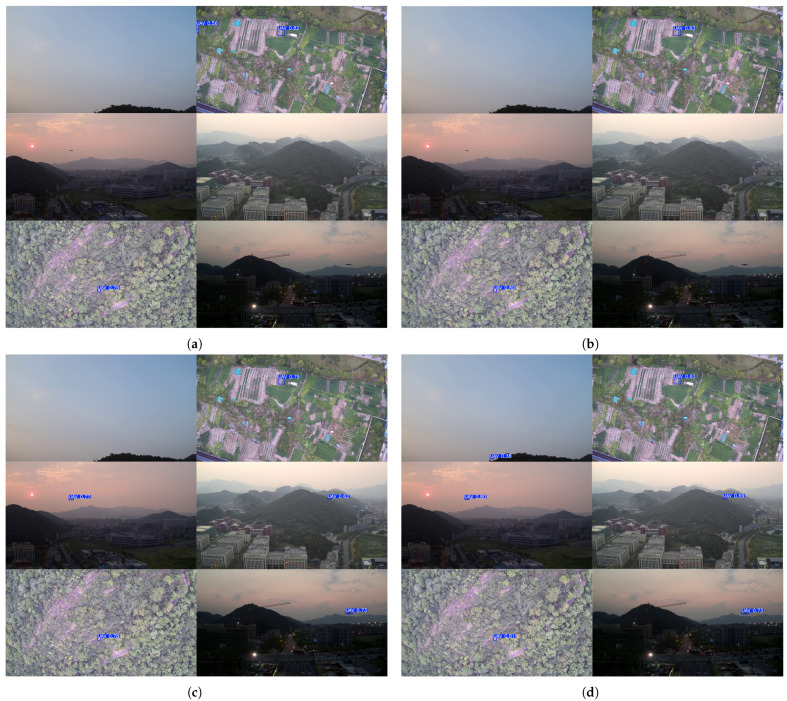
Comparison of UAV detection outcomes in complex background scenarios. The blue boxes indicate the model-predicted bounding boxes of UAV targets. (**a**) YOLOv5n; (**b**) YOLOv8n; (**c**) YOLOv11n; (**d**) proposed SPAE-YOLOv8.

To intuitively demonstrate the performance benefits of the SPAE-YOLOv8 algorithm for air-to-air small UAV target detection, representative samples featuring complex urban backgrounds, small targets, and varying viewing angles were selected from the Det-Fly dataset. Comparative visual experiments were carried out among SPAE-YOLOv8, YOLOv5n, YOLOv8n, and YOLOv11n, with the resulting detection outputs illustrated in Figure 8.

As shown in Figure 8, when UAV targets appear as small objects and their colors are similar to the background, the YOLOv5n model only detects part of the distinguishable UAV targets, and in particular, false detection occurs in the second subfigure of Figure 8a; in Figure 8b, the YOLOv8n model improves detection accuracy and reduces false detections in both images, but partial false detections still exist under low-light and background-similar scenarios; in Figure 8c, the YOLOv11n model alleviates target missed detection, yet some small targets remain undetected, whereas the improved SPAE-YOLOv8 model can accurately identify small UAV targets and those under complex backgrounds, achieving excellent detection performance with further improved accuracy even in poor lighting conditions; unlike YOLOv8n, which misses many small targets, our model successfully detects small UAVs and those in complex backgrounds, thereby demonstrating its capability to effectively detect small UAVs in air-to-air scenarios, and the improved model not only correctly identifies target categories with higher accuracy but also performs well in low-light environments, demonstrating its adaptability to different environmental conditions.

### 4.7. Cross-Dataset Performance Validation

To comprehensively evaluate the performance and stability of the SPAE-YOLOv8 algorithm across various scenarios and data distributions, in addition to the Det-Fly dataset, this section conducts experiments on the publicly available DUT Anti-UAV visible-light dataset provided by Dalian University of Technology. This dataset [29] is designed specifically for single-frame anti-UAV detection and contains 10,000 images representing complex outdoor real-world scenes. The dataset is divided into training, validation, and test subsets with 5200, 2600, and 2200 images, respectively, and includes a total of 10,109 annotated UAV targets. Image resolutions range from 160×240 to 3744×5616, encompassing diverse backgrounds such as urban buildings, jungles, farmland, and sky, along with varied illumination and weather conditions including day, night, dawn/dusk, sunny, and cloudy. The dataset primarily consists of extremely small-scale UAV targets, with an average target area ratio of approximately 0.013. It also presents challenges such as large scale differences, varying aspect ratios, and significant background interference, which provide valuable experimental data for evaluating algorithm performance in real-world anti-UAV detection scenarios.

In the following experiments, consistent training configurations and hardware environments with those used on the Det-Fly dataset are maintained, and training is performed for 200 epochs. Using the evaluation metrics introduced earlier, the proposed algorithm is compared with representative one-stage, two-stage, and end-to-end detection models to further evaluate its robustness and speed–accuracy trade-off under different data distributions. The corresponding experimental results are summarized in Table 8.

As presented in Table 8, to validate the cross-dataset generalization ability of the proposed SPAE-YOLOv8, we compare it with several representative object detection models, including YOLO series models, Hyper-YOLO, Drone-YOLO, Faster R-CNN, and RT-DETR-R18. Among them, YOLO series models are classic and widely used real-time detectors that achieve a favorable balance between accuracy and speed. Hyper-YOLO enhances feature representation capability through advanced network design, while Drone-YOLO is an excellent model specifically designed for drone object detection.

For the newly added non-YOLO baselines, Faster R-CNN shows limited suitability for the DUT Anti-UAV dataset. It achieves only 0.683 mAP@0.5 and 0.328 mAP@0.5:0.95, while requiring 28.3 M parameters and a model size of 108.2 MB. Its inference speed is only 34.0 FPS, indicating that the two-stage detection paradigm has relatively high computational and storage costs for lightweight onboard deployment. In contrast, RT-DETR-R18 achieves the highest mAP@0.5 of 0.923 and mAP@0.5:0.95 of 0.609, showing strong detection accuracy on the DUT Anti-UAV dataset. However, it requires 19.9 M parameters and a model size of 38.6 MB, and its inference speed is only 103.8 FPS. Therefore, although RT-DETR-R18 provides high detection accuracy, its heavier model scale and lower inference speed make it less suitable for resource-constrained micro-UAV onboard deployment.

**Figure 9:**
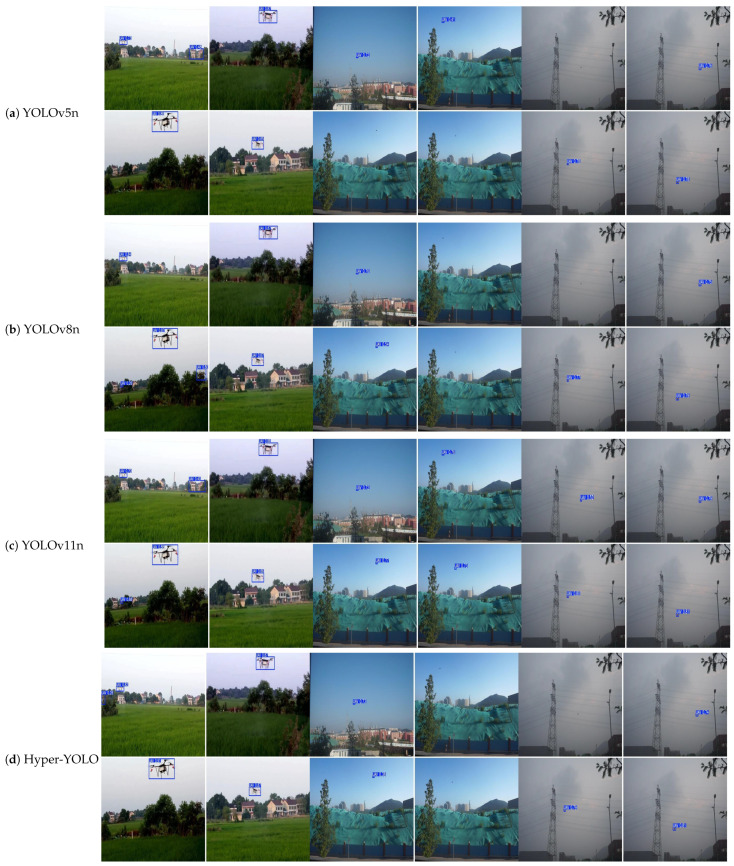

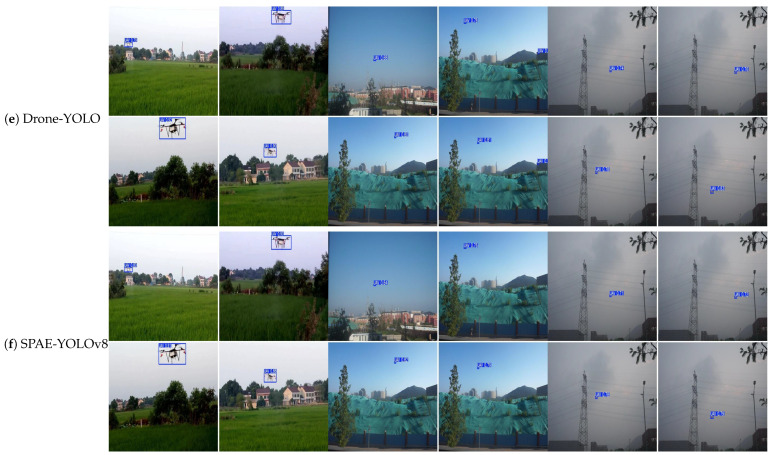
Visual comparison of detection results on the DUT Anti-UAV dataset under various air-to-air scenarios. Each row corresponds to one detector, showing 12 typical test images under different lighting conditions and target scales. The blue boxes indicate the model-predicted bounding boxes of UAV targets.

Experimental results on the DUT Anti-UAV dataset demonstrate that SPAE-YOLOv8 still achieves strong comprehensive performance. It attains the highest precision of 0.966, and obtains competitive recall (0.850), mAP@0.5 (0.906), and mAP@0.5:0.95 (0.593), showing strong robustness against complex backgrounds and micro-drone targets. Meanwhile, with only 2.1 million parameters and a model size of 4.3 MB, SPAE-YOLOv8 achieves the highest inference speed of 196.9 FPS among all compared models, making it more lightweight and efficient than the baseline models. Its Efficiency Score reaches 84.95, which is substantially higher than Faster R-CNN, RT-DETR-R18, Drone-YOLO, and other YOLO variants, further demonstrating its favorable speed–accuracy–complexity trade-off under cross-dataset validation. The model achieves significant improvements in both detection accuracy and inference efficiency, making it more suitable for real-time and lightweight deployment in practical anti-drone systems. To visually demonstrate the detection performance of our SPAE-YOLOv8 model, we present visual results in Figure 9.

To further compare the detection performance of our algorithm, we visually present partial detection results of six advanced detectors on the DUT Anti-UAV dataset. All test images are obtained from the independent test set of the dataset and do not participate in model training or validation parameter tuning, ensuring fair and reliable experimental results. To demonstrate detection performance under as many diverse backgrounds as possible, typical results are arranged as follows: each row corresponds to one detector, each row contains three subfigures, and each subfigure shows four images of the same UAV target at different time frames. Thus, each row contains 12 valid test images covering farmland, sky, power lines, and other complex outdoor scenes.

In the first type of farmland background, the UAV is surrounded by crops and buildings, which cause strong interference. YOLOv5n, YOLOv8n, YOLOv11n, and Hyper-YOLO exhibit different degrees of false detection. Only Drone-YOLO and the proposed SPAE-YOLOv8 can accurately detect the target with high confidence, indicating that the proposed algorithm effectively accomplishes small and medium target detection in complex backgrounds.

In the second type of aerial background, the UAV target is extremely small. Most algorithms, including YOLOv8n and Drone-YOLO, suffer from missed or false detections. Only YOLOv11n and SPAE-YOLOv8 can accurately identify the target with a confidence level above 0.7, without any false or missed detections.

In the third type of power line background, the UAV target is tiny and the background interference is intense. The proposed SPAE-YOLOv8 still detects all targets accurately. In addition, our algorithm achieves excellent performance in various other complex outdoor backgrounds.

Meanwhile, the proposed algorithm reduces the model parameter count and improves inference speed, rendering it more suitable for air-to-air small UAV detection tasks and granting it greater practical application potential, which intuitively demonstrates its strong generalization ability across different datasets.

### 4.8. Cross-Dataset Validation on Anti-UAV300

To strengthen the generalization claim of the proposed model, we conduct further validation on a third and more challenging public dataset, i.e., Anti-UAV (also known as Anti-UAV300). Released by the Visual Geometry Group at the University of Chinese Academy of Sciences, this dataset consists of 318 high-definition video sequences with more than 580,000 high-precision annotations, covering typical challenging attributes in air-to-air scenarios including fast motion, occlusion, low illumination, and low resolution. Different from the two original datasets with relatively clean backgrounds, Anti-UAV300 contains complex background interference and obvious image degradation, which is more in line with the actual complex air-to-air detection environment. To ensure experimental consistency, we only adopt the RGB visible-light modality of Anti-UAV300 and construct static image samples by extracting one frame every 20 frames from original video sequences. Finally, 5104 training images and 2209 validation images are obtained for cross-dataset evaluation.

**Figure 10:**
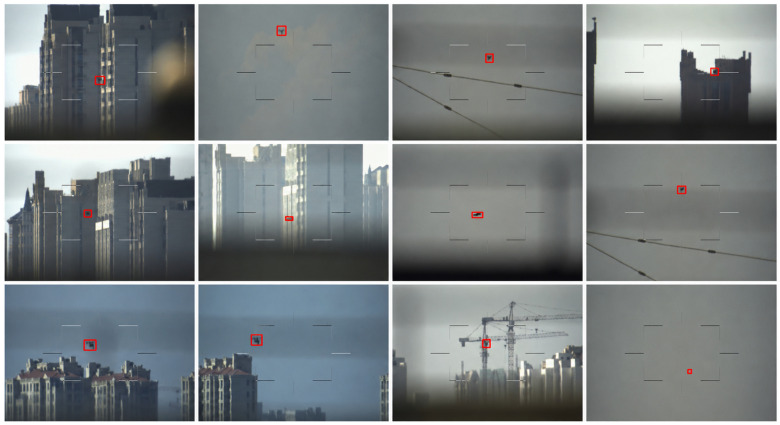
Sample images of the Anti-UAV300 dataset (with ground-truth bounding boxes for intuitive display). The red boxes indicate the ground-truth bounding boxes of UAV targets, while the black and white lines are reticle guide lines from the original imaging interface rather than additional annotations.

In the cross-dataset validation setting, the model fully trained on the DUT Anti-UAV training set is directly deployed for inference on the Anti-UAV300 validation set without any additional fine-tuning or parameter adaptation. Figure 10 illustrates representative image samples and corresponding annotation bounding boxes from the Anti-UAV300 validation set. As depicted, the dataset includes various difficult scenarios such as tiny drone targets, cluttered background textures, dim lighting and partial occlusion, which intuitively reflects its higher detection difficulty and scenario diversity. It further demonstrates the rationality and necessity of adopting this dataset to verify the generalization ability of the proposed method.

To objectively evaluate the cross-dataset generalization capability, we only conduct a comparative test between the baseline YOLOv8n and our proposed SPAE-YOLOv8 under the unified experimental configuration. Both models are trained on the DUT Anti-UAV dataset and directly evaluated on the Anti-UAV300 validation set without extra fine-tuning, guaranteeing a fair and consistent comparison condition.

Table 9 summarizes the quantitative evaluation results of the baseline YOLOv8n and the proposed SPAE-YOLOv8 on the Anti-UAV300 dataset, including key metrics such as Precision, Recall, mAP@0.5, inference latency, FPS, model parameters, model size, and Efficiency Score. It can be observed that, benefiting from the optimized feature extraction and small-target enhancement design, the proposed SPAE-YOLOv8 achieves a better comprehensive balance between detection accuracy, real-time performance, and lightweight capability compared with the baseline YOLOv8n. Specifically, our model maintains competitive Precision and Recall while having fewer parameters and an acceptable inference latency, which meets the real-time constraints of micro-UAV airborne detection. The higher Efficiency Score further confirms the superior overall performance of our method, and the cross-dataset evaluation results fully demonstrate that the proposed model possesses stronger generalization and robustness in unseen, complex real-world aerial detection scenarios.

### 4.9. Real-World Experiment

To further validate the generalization performance of the SPAE-YOLOv8 model trained on the Det-Fly dataset, real-world onboard deployment and real-time detection experiments are conducted using a self-developed UAV platform. The UAV equipped with an onboard computer performs real-time detection on targets captured in practical scenarios, and the recorded video data during detection is simultaneously transmitted back to the cloud, thereby demonstrating the engineering application value of the model in real air-to-air scenarios.

**Figure 11:**
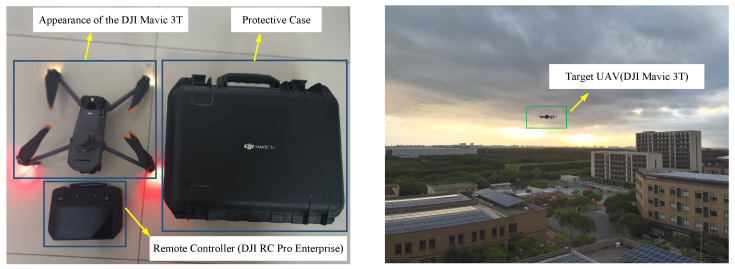
Target drone: DJI Mavic 3T, appearance and aerial view images.

In the real-world generalization experiment, a DJI Mavic 3T UAV was chosen as the target aircraft. Images of the UAV were collected under practical observation conditions and used for inference to examine the cross-scene generalization ability of the model beyond public benchmark datasets. This experiment aims to confirm whether the detector, trained for air-to-air small target detection, can still achieve reliable target recognition in real environments where background and imaging conditions differ from the training data. This validation aligns with the design goal of the proposed method, which is to enhance YOLOv8n’s applicability for air-to-air small target detection while retaining lightweight characteristics. The target UAV is shown in Figure 11.

**Figure 12:**
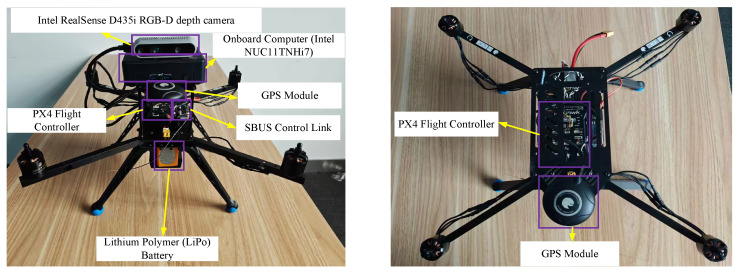
Intel NUC11TNHi7-Based Quadrotor UAV Platform.

For onboard deployment, a custom quadrotor UAV platform was constructed, as shown in Figure 12. The platform consists of a self-assembled four-rotor airframe equipped with an Intel NUC11TNHi7 mini PC (Intel Corporation, Santa Clara, CA, USA) for onboard computation. The computer is powered by an Intel Core i7-1165G7 processor (Intel Corporation, Santa Clara, CA, USA) (4 cores and 8 threads) and Intel Iris Xe integrated graphics (Intel Corporation, Santa Clara, CA, USA), representing a typical resource-constrained airborne embedded computing environment.

To further clarify the hardware requirements of the proposed model, the onboard deployment configuration is summarized in Table 10. It should be noted that the proposed SPAE-YOLOv8 model does not require a discrete GPU during onboard inference. In our real-world deployment, the model was executed on an Intel NUC11TNHi7 mini PC equipped with an Intel Core i7-1165G7 processor and Intel Iris Xe integrated graphics. With only 2.1 M parameters and a 4.3 MB model size, the proposed model has low storage and computational requirements. Therefore, the model is compatible with resource-constrained edge devices and is suitable for micro-UAV onboard perception.

During deployment, the trained SPAE-YOLOv8 model was first exported to ONNX format and then optimized using OpenVINO for onboard inference acceleration. This deployment strategy also aligns with the lightweight design goal of the proposed method, since the model was specifically developed for airborne embedded scenarios and highlights the comprehensive optimization of detection precision and inference efficiency. In previous comparative experiments, SPAE-YOLOv8 achieved strong detection performance with merely 2.1 M parameters alongside a 4.3 MB model size, further indicating its suitability for edge-side deployment.

**Figure 13:**
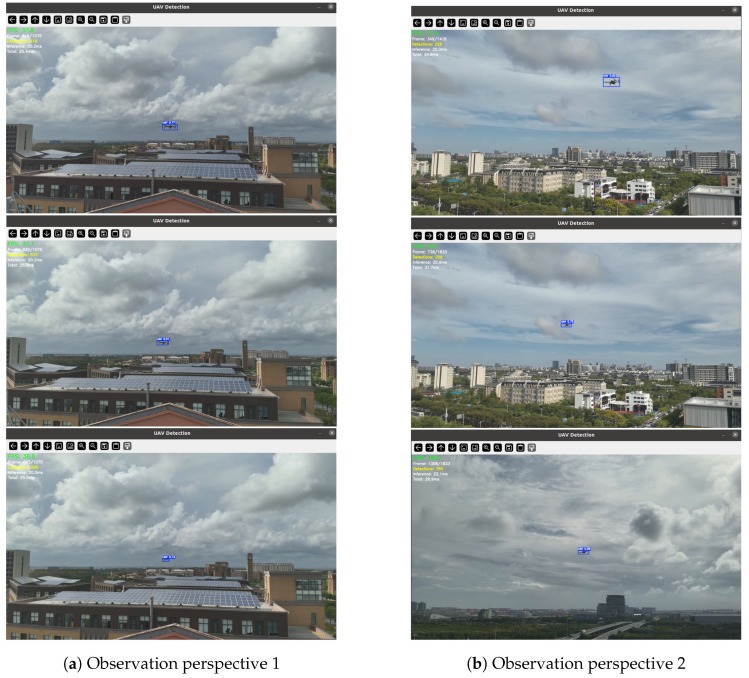
Experimental results for real-world air-to-air UAV object detection from two observation perspectives of the same target UAV. The blue boxes indicate the model-predicted bounding boxes of UAV targets.

The real-world experimental findings indicate that the proposed model can still accurately detect the DJI Mavic 3T target under practical imaging conditions, demonstrating good generalization ability outside the training dataset. Meanwhile, after OpenVINO-based deployment on the Intel NUC11TNHi7 onboard computer (Intel Corporation, Santa Clara, CA, USA), the inference speed of the model reached 43.9 FPS at 640 × 640 resolution, corresponding to an average detection latency of 22.8 ms per image. As shown in Figure 13, the instantaneous inference time and total processing time fluctuate slightly across frames, where the total time additionally includes visualization and display overhead. These results indicate that the proposed method can process each image within a short delay and satisfies the real-time detection requirement for micro-UAV onboard applications.

In addition, Figure 13 visualizes the detection performance of the proposed method in typical air-to-air scenes. The left subgraph (a) presents observation perspective 1 of the single UAV, while the right subgraph (b) illustrates observation perspective 2 of the same UAV. These intuitive visualization results demonstrate that our model can accurately detect UAV targets in real time. It maintains stable detection performance under changing viewing angles and complex interference conditions, further verifying the strong robustness and practical application value of the proposed method for challenging air-to-air detection tasks.

Overall, the real-world generalization and onboard deployment experiments further verify that the enhanced SPAE-YOLOv8 model yields superior performance on public air-to-air UAV datasets, while demonstrating excellent deployment potential and practical application value on real-world UAV platforms. The experimental results fully demonstrate that the proposed method can maintain a favorable balance among detection accuracy, model lightweightness, and real-time inference speed.

## 5. Conclusions

This study addressed the challenge of achieving accurate, lightweight, and real-time air-to-air small UAV detection under onboard deployment constraints. Based on YOLOv8n, the SPAE-YOLOv8 model was developed by integrating the SIoU loss function, the P2 shallow feature layer, the ADown refined downsampling module, and the Efficient_UAVDet lightweight detection head. These modules were designed to improve small-target representation, reduce downsampling-related information loss, enhance localization accuracy, and decrease detection-head redundancy.

The experimental results on the Det-Fly dataset showed that SPAE-YOLOv8 achieved an mAP@0.5 of 0.922 and an mAP@0.5:0.95 of 0.582, improving mAP@0.5 by 7.2 percentage points compared with the YOLOv8n baseline. Meanwhile, the model parameters were reduced to 2.1 M and the model size was reduced to 4.3 MB, indicating that the proposed architecture achieved a better balance between detection accuracy and model lightweightness. The ablation studies further showed that the final improvement was produced by the complementary effects of different modules rather than by a single component alone.

Independent training and testing on the DUT Anti-UAV dataset further verified the robustness of SPAE-YOLOv8 under different data distributions and complex outdoor backgrounds. In addition, cross-dataset validation was conducted on the more challenging Anti-UAV300 dataset, where models trained on DUT Anti-UAV were directly evaluated without additional fine-tuning. The results demonstrated that the proposed method maintained better generalization ability under challenging conditions such as fast motion, occlusion, low illumination, low resolution, cluttered backgrounds, and image degradation. The onboard deployment experiment also verified the practical feasibility of the proposed method. After OpenVINO-based acceleration on the Intel NUC11TNHi7 embedded platform, SPAE-YOLOv8 achieved 43.9 FPS at 640 × 640 resolution, corresponding to an average detection latency of 22.8 ms per image. This result indicated that the proposed model could satisfy the real-time inference requirements of micro-UAV onboard perception while maintaining a compact model structure.

Although the proposed method achieved promising results, several limitations remained. The current real-world validation was mainly conducted under visible-light conditions, and the robustness of the model under more extreme environments still requires further investigation. It should also be noted that the current real-world onboard validation mainly used a DJI Mavic 3T as the target UAV. Therefore, the cross-platform generalization capability of the proposed method for heterogeneous UAV types, such as fixed-wing UAVs and racing drones, still requires further validation. Future work will focus on expanding real-world flight experiments under adverse weather, severe illumination changes, stronger motion blur, and more diverse UAV platforms with different airframe structures and motion characteristics. In addition, infrared or thermal imaging modalities will be considered to improve detection robustness under low-light, nighttime, and complex background conditions.

## Figures and Tables

**Table 1:** Summary of existing small-target and lightweight UAV detection methods.

Reference	Method	Core Idea	Limitation
[11]	YOLOv8 + Self-Attention	Pruned layers + attention head	Ground-object focus
[12]	TFP-YOLO	Triplet attention + TFE + P2	Road-scene task
[13]	Improved YOLOv8n	Extra heads + CondConv + Wise-IoU	Limited air-to-air validation
[14]	SOD-YOLO	RFCBAM + BSSI-FPN	Complex fusion structure
[15]	SS-YOLOv8	Tiny-object head + SPD-Conv + GCU	UAV-view ground targets
[16]	UAV-YOLOv8	WIoUv3 + BiFormer + FFNB	Limited for textureless objects
[17]	MobileNet	Depthwise separable convolution	Weak feature representation
[18]	ShuffleNet	Group convolution + channel shuffle	Generic classification backbone
[19]	GhostNet	Cheap feature generation	Limited small-target localization
[20]	GCL-YOLO	GhostConv + small-object head	Ground-object UAV data
[21]	YOLO-SDH	Scaled decoupled head + deformable conv	Not UAV-specific
[22]	EfficientDet	BiFPN + compound scaling	Generic detection framework
[23]	DynamicHead	Multi-dimensional head attention	Not lightweight-oriented
YOLOv8n	Baseline detector	Lightweight and fast inference	Weak tiny-UAV detection

**Table 2:** Comparison of representative UAV detection datasets.

Dataset	Perspective	Target Characteristic	Limitation
Aircraft [24]	Air-to-air	Small flying objects	Not open-source
Det-Fly [25]	Air-to-air	Tiny micro UAV (0.12%)	Only one UAV type
Avian-Airborne [26]	Air-to-air	Aircraft, birds, UAVs	Small scale, limited scene diversity
ARD100 [27]	Air-to-air	Tiny UAV, RGB + motion	Only quadrotors, no fixed-wing
MMFW-UAV [28]	Air-to-air	Fixed-wing UAV, multi-sensor	Fixed-wing only, no quadrotors
DUT Anti-UAV [29]	Air-to-air	UAV detection, complex background	RGB-only, no multi-modal
Anti-UAV300 [30]	Air-to-air	UAV tracking, occlusion, blur	Mostly sky background
UAVid [31]	Air-to-ground	Semantic segmentation of ground scenes	Not designed for object detection
AU-AIR [32]	Air-to-ground	Multi-modal low-altitude traffic data	Focuses on ground vehicles/pedestrians
VisDrone [33]	Air-to-ground	Dense ground objects from aerial view	Not for air-to-air UAV detection

**Table 3:** Channel-adaptive group convolution configuration for detection head branches.

Detection Head Branch	Input Channels *x*	Group Number *g*	Channels per Group x/g
P2	32	2	16
P3	64	4	16
P4	128	8	16
P5	256	16	16

Note: *x* denotes the input channel number of each detection head branch, *g* denotes the number of groups, and x/g denotes the number of channels assigned to each group.

**Table 4:** Training parameter configuration.

Parameter	Setting
optimizer	SGD
epochs	200
batch	32
workers	4
imgsz	640
close_mosaic	10
warmup_momentum	0.8
warmup_epochs	3
momentum	0.937
weight_decay	0.0005
lrf	0.01
lr0	0.01

**Table 5:** Results of experiments comparing methods on the Det-Fly dataset.

Models	Precision	Recall	mAP@ 0.5	mAP@ 0.5:0.95	FPS (f/s)	Parameters ($10^{6}$)	Model Size (MB)	Efficiency Score
Faster R-CNN [45]	0.472	0.753	0.705	0.326	35.9	28.3	108.2	0.90
YOLOv5n [46]	0.933	0.774	0.842	0.504	190.8	2.5	5.0	64.26
YOLOv8n [47]	0.941	0.780	0.850	0.529	197.2	3.0	5.9	55.87
YOLOv10n [48]	0.926	0.768	0.860	0.543	195.7	2.3	5.5	73.17
YOLOv11n [49]	0.939	0.872	0.904	0.548	193.1	2.6	5.2	67.14
Drone-YOLO [50]	0.952	0.890	0.927	0.573	160.5	3.0	6.0	49.59
RT-DETR-R18 [51]	0.975	0.904	0.941	0.620	110.8	19.9	38.6	5.24
SPAE-YOLOv8 (This approach)	0.954	0.881	0.922	0.582	203.0	2.1	4.3	89.13

**Table 6:** Ablation experimental results on the Det-Fly dataset.

Baseline	SIoU	P2	Adown	Efficient_ UAVDet	Precision	Recall	mAP@ 0.5	mAP@ 0.5:0.95	FPS (f/s)	Parameters ($10^{6}$)	Model Size (MB)
✓					0.941	0.780	0.850	0.529	197.2	3.0	5.9
✓	✓				0.930	0.792	0.851	0.517	198.0	3.0	5.9
✓		✓			0.953	0.886	0.925	0.576	168.9	2.9	5.9
✓			✓		0.946	0.780	0.854	0.524	187.2	2.6	5.4
✓				✓	0.931	0.780	0.846	0.521	252.9	2.4	4.8
✓	✓	✓			0.950	0.887	0.929	0.589	170.8	2.9	5.9
✓	✓		✓		0.935	0.787	0.851	0.525	185.9	2.6	5.2
✓			✓	✓	0.925	0.785	0.850	0.519	237.3	2.0	4.0
✓		✓	✓		0.955	0.875	0.922	0.586	162.6	2.5	5.1
✓		✓		✓	0.947	0.881	0.920	0.577	217.0	2.5	5.0
✓	✓			✓	0.923	0.786	0.851	0.523	256.1	2.4	4.8
✓		✓	✓	✓	0.948	0.882	0.921	0.578	199.0	2.1	4.3
✓	✓		✓	✓	0.935	0.778	0.849	0.520	236.6	2.0	4.0
✓	✓	✓		✓	0.949	0.884	0.918	0.573	218.3	2.5	5.0
✓	✓	✓	✓		0.961	0.882	0.929	0.593	161.5	2.5	5.1
✓	✓	✓	✓	✓	0.954	0.881	0.922	0.582	203.0	2.1	4.3

Note: ✓ indicates that the corresponding module is used in the model configuration.

**Table 7:** Sensitivity analysis of different channel-per-group settings in Efficient_UAVDet on the Det-Fly dataset.

Channels per Group x/g	Group Numbers P2/P3/P4/P5	Precision	Recall	mAP@ 0.5	mAP@ 0.5:0.95	FPS (f/s)	Parameters ($10^{6}$)	GFLOPs	Model Size (MB)
8	4/8/16/32	0.956	0.876	0.922	0.580	201.1	1.99	6.0	4.1
16	2/4/8/16	0.954	0.881	0.922	0.582	203.0	2.06	6.4	4.3
32	1/2/4/8	0.954	0.875	0.918	0.579	196.4	2.19	7.3	4.5

Note: x/g denotes the number of channels assigned to each group. The group numbers correspond to the P2, P3, P4, and P5 detection heads, respectively.

**Table 8:** Comparative experimental results on the DUT Anti-UAV dataset.

Models	Precision	Recall	mAP@ 0.5	mAP@ 0.5:0.95	FPS (f/s)	Parameters ($10^{6}$)	Model Size (MB)	Efficiency Score
Faster R-CNN [45]	0.472	0.720	0.683	0.328	34.0	28.3	108.2	0.82
YOLOv5n [46]	0.936	0.725	0.811	0.509	189.6	2.5	5.0	61.53
YOLOv8n [47]	0.935	0.738	0.826	0.526	191.3	3.0	6.0	52.62
YOLOv10n [48]	0.921	0.760	0.846	0.543	191.9	2.3	5.5	70.48
YOLOv11n [49]	0.952	0.818	0.877	0.548	193.2	2.6	5.2	65.32
YOLOv12n [52]	0.932	0.724	0.816	0.518	178.3	2.5	5.2	58.23
Hyper-YOLO [53]	0.936	0.768	0.844	0.542	148.3	3.9	7.9	32.22
Drone-YOLO [50]	0.959	0.847	0.907	0.594	166.7	3.0	6.1	50.33
RT-DETR-R18 [51]	0.964	0.903	0.923	0.609	103.8	19.9	38.6	4.82
SPAE-YOLOv8 (This approach)	0.966	0.850	0.906	0.593	196.9	2.1	4.3	84.95

**Table 9:** Cross-dataset comparison results on the Anti-UAV300 dataset.

Models	Precision	Recall	mAP@ 0.5	Latency (ms)	FPS (f/s)	Parameters ($10^{6}$)	Model Size (MB)	Efficiency Score
YOLOv8n [47]	0.922	0.542	0.670	5.2	191.3	3.01	6.0	42.60
SPAE-YOLOv8 (This approach)	0.930	0.557	0.705	4.9	202.3	2.06	4.3	69.25

**Table 10:** Hardware configuration and deployment requirements for onboard inference.

Item	Configuration/Requirement
Deployment platform	Intel NUC11TNHi7 onboard mini PC
Processor	Intel Core i7-1165G7, 4 cores/8 threads
Graphics unit	Intel Iris Xe integrated graphics
Discrete GPU	Not required for onboard inference
Acceleration framework	OpenVINO
Model format	ONNX/OpenVINO-optimized model
Input resolution	640×640
Model parameters	2.1 M
Model size	4.3 MB
Average speed	43.9 FPS
Average latency	22.8 ms/image

## Data Availability

The Det-Fly dataset employed in this work is publicly accessible at https://github.com/Jake-WU/Det-Fly (accessed on 19 April 2026). This repository further provides the corresponding download links for all image data and annotation files, as detailed in Reference [25]. Meanwhile, the DUT Anti-UAV dataset adopted in this study is publicly available at https://github.com/wangdongdut/DUT-Anti-UAV (accessed on 19 April 2026), which contains complete image and annotation resources, as cited in Reference [29]. The Anti-UAV300 dataset used for cross-dataset validation is publicly available at https://github.com/ZhaoJ9014/Anti-UAV (accessed on 19 April 2026), as described in Reference [30].
